# Diagnostic performance of a 5-plex malaria immunoassay in regions co-endemic for *Plasmodium falciparum*, *P. vivax*, *P. knowlesi*, *P. malariae* and *P. ovale*

**DOI:** 10.1038/s41598-022-11042-w

**Published:** 2022-05-04

**Authors:** Steven Kho, Nicholas M. Anstey, Bridget E. Barber, Kim Piera, Timothy William, Enny Kenangalem, James S. McCarthy, Ihn Kyung Jang, Gonzalo J. Domingo, Sumudu Britton, Matthew J. Grigg

**Affiliations:** 1 Menzies School of Health Research and Charles Darwin University, Darwin, NT Australia; 2 QIMR-Berghofer Medical Research Institute, Brisbane, QLD Australia; 3 Clinical Research Centre, Queen Elizabeth Hospital, Kota Kinabalu, Malaysia; 4 Papuan Health and Community Development Foundation, Timika, Indonesia; 5 The Peter Doherty Institute for Infection and Immunity, University of Melbourne and Royal Melbourne Hospital, Melbourne, VIC Australia; 6 Diagnostic Program, PATH, Seattle, USA

**Keywords:** Infectious-disease diagnostics, Malaria

## Abstract

Commercial point-of-care tests remain insufficient for accurately detecting and differentiating low-level malaria infections in regions co-endemic with multiple non-falciparum species, including zoonotic *Plasmodium knowlesi* (Pk). A 5-plex chemiluminescent assay simultaneously measures pan-*Plasmodium* lactate dehydrogenase (pLDH), *P. falciparum* (Pf)-LDH, *P. vivax* (Pv)-LDH, Pf-histidine-rich protein-2 (HRP2), and C-reactive protein. We assessed its diagnostic performance on whole blood (WB) samples from 102 healthy controls and 306 PCR-confirmed clinical cases of Pf, Pv, Pk, *P. malariae* (Pm) and *P. ovale* (Po) mono-infections from Southeast-Asia. We confirm its excellent HRP2-based detection of Pf. Cross-reactivity of Pf-LDH with all non-falciparum species tested was observed (specificity 57.3%). Pv-LDH performance was suboptimal for Pv (93.9% sensitivity and 73.9% specificity). Poor specificity was driven by strong Pk cross-reactivity, with Pv-LDH detecting 93.9% of Pk infections. The pan-LDH-to-Pf-LDH ratio was capable of discerning Pv from Pk, and robustly differentiated Pf from Pm or Po infection, useful in regions with *hrp2/3* deletions. We tested the platform’s performance in plasma for the first time, with WB outperforming plasma for all analytes except Pv-LDH for Pk. The platform is a promising tool for WB malaria diagnosis, although further development is warranted to improve its utility in regions co-endemic for multiple non-falciparum species.

## Introduction

Malaria remains a major global problem, with 241 million cases and 627,000 deaths reported in 2020^1^. Ongoing challenges in malaria elimination include the lack of adequate tools to detect the low parasite densities seen in asymptomatic infection, with this group serving as a significant reservoir for transmission^2,3^. Light microscopy and rapid diagnostic tests (RDTs), the most commonly used point-of-care malaria diagnostics in endemic areas, lack sensitivity in low-level infection, and often demonstrate poor accuracy for differentiating non-falciparum species^4,5^. The development of highly sensitive and specific malaria diagnostic tools is essential both for effective treatment and for prevention of onward transmission^6^.

A 5-plex chemiluminescent enzyme-linked immunosorbent assay (ELISA) (Quansys Biosciences) simultaneously detects and quantifies histidine-rich protein-2 (HRP2), *Plasmodium falciparum* lactate dehydrogenase (Pf-LDH), *P.* *vivax*-LDH (Pv-LDH), pan-*Plasmodium-*LDH (pan-LDH) and C-reactive protein (CRP), a host inflammatory marker^7^. The performance of 4-plex^8^ and 5-plex^7^ assays for antigen-based malaria screening has been tested on whole blood from asymptomatic and symptomatic infections with *P. falciparum*, *P. vivax*, *P. malariae* and *P. ovale* and on matching dry blood-spots^9^, reporting high sensitivity for *P. falciparum*, including for those with *hrp2/3* deletions. Apart from *P. cynomolgi*^10^, genus-specific pLDH has enabled detection of all human *Plasmodium* species in whole blood with varying sensitivity. However, because of the high level of conservation of the *Plasmodium* protein, anti-pLDH antibodies designed to detect species-specific epitopes in the protein have demonstrated cross-reactivity with other species, resulting in loss of specificity^10^.

*Plasmodium knowlesi* causes zoonotic malaria across most of Southeast Asia, including severe and fatal disease^11,12^. Knowlesi malaria is co-endemic with *P. falciparum*, *P. vivax*, *P. malariae* and *P. ovale* across much of its geographical range. Because of high levels of conservation in antigenic targets between *P. knowlesi* and other non-falciparum species, especially *P. vivax*, there is significant cross-reactivity between species^13–16^. RDTs evaluated to date have had low sensitivity and poor specificity for diagnosis of *P. knowlesi*^17–19^. As a zoonosis, its epidemiology and strategies for control differ significantly from other species causing malaria, making accurate diagnosis important. The 5-plex Quansys platform has significant cross-reactivity between Pv-LDH and *P. knowlesi* generated from *in-vitro* cultures^10^. Detection of low parasitemia with high specificity and sensitivity for all *Plasmodium* species causing human malaria in co-endemic areas is essential.

Here, we evaluated the diagnostic performance of the 5-plex platform in whole blood and for the first time in plasma from malaria patients in regions co-endemic for the five major human *Plasmodium* species, and provide novel data for clinical *P. knowlesi* infections.

## Methods

### Samples and study procedures

Clinical malaria cases from two endemic regions in Southeast Asia were included in the study. Peripheral venous blood was collected from malaria patients presenting to health facilities with fever and infected with *Plasmodium falciparum, P. vivax, P. knowlesi, P. malariae* or *P. ovale* spp. as part of prospective clinical studies in Sabah, Malaysia^20,21^. Venous blood was also concurrently collected from healthy asymptomatic adult controls negative for malaria on microscopy and subsequent polymerase chain reaction (PCR) testing^22^. At the time of sampling Sabah had co-endemic transmission of the five aforementioned *Plasmodium* species. Additional samples from a small number of patients with *P. ovale* infection were obtained from a clinical trial in Timika^23^, a lowland forest region of Indonesian Papua with very low prevalence of this species^24^. Malaria patients in these regions were enrolled according to inclusion and exclusion criteria as previously described^20,21,25^. Clinical and laboratory features to define severe malaria were based on WHO 2014 research criteria^21,26^. Whole blood and plasma samples were stored at − 80 °C within 30 min and thawed prior to antigen evaluations. *Plasmodium* species mono-infections were confirmed for all malaria cases by PCR using validated protocols described elsewhere^27,28^. Parasite counts per 200 white cells (thick smear) or 1,000 red cells (thin smear) were determined by experienced research microscopists, then converted to parasites per microlitre blood using automated cell counts.

### Multiplex ELISA

The commercially-available Q-Plex™ Human Malaria assay (Quansys Biosciences, Logan, UT, USA) was used to detect and quantify four *Plasmodium* antigens and CRP in ethylenediaminetetraacetic acid-anticoagulated whole blood and heparinised plasma. The assay was performed according to manufacturer’s instructions and serially diluted protein standards from the kit were used to construct standard curves. Chemiluminescent imaging of each plate was captured on a Q-View™ Imager LS and analysed using Q-View™ software (Quansys Biosciences). Samples were run neat and diluted up to 100-fold until results were obtained in a reliable area of the standard curves. Several samples needing further dilution for HRP2 and CRP were assigned a value of the upper limit of quantification on their standard curve. Samples run neat that had antigen(s) below the detection limit were conservatively assigned a value corresponding to the assay’s lower limit of detection for that antigen(s).

### Statistical analysis

Statistical analyses were performed using Graphpad Prism 9.2.0 (San Diego, CA, USA). The Mann–Whitney test was used for the comparison of two patient groups, and the Kruskal–Wallis test with Dunn’s multiple comparison was used for comparisons with > 2 groups. To evaluate diagnostic sensitivity and specificity, the PCR *Plasmodium* species result was used as the gold-standard reference. For the Q-Plex™ Human Malaria assay, the mean plus 3 × standard deviation of the control group was then used as the threshold cut-off to determine positivity for each antigen. Two other standard methods to determine threshold cut-offs were used as additional comparators – values giving > 99% specificity on receiver-operating-characteristic (ROC) curves versus (a) controls and (b) non-cognate *Plasmodium* species ^7^. Sensitivity was calculated as true positives divided by the sum of true positives and false negatives. Specificity was calculated as true negatives divided by the sum of true negatives and false positives. These values were expressed as percentages and reported with 95% confidence intervals (CI) determined using the binomial exact method. The McNemar paired chi-squared test with Yates correction was used for the comparison of assay sensitivities between paired plasma and whole blood.

### Ethical approval

Studies were approved by the Human Research Ethics Committees of the Ministry of Health, Malaysia; the National Institute of Health Research and Development, Ministry of Health, Indonesia; and Menzies School of Health Research, Northern Territory, Australia. Written informed consent was obtained from all participants or their legal guardian. All methods were performed in accordance with relevant guidelines and regulations.

## Results

### Demographic data

A total of 102 healthy controls and 306 malaria patients were studied, comprising the following PCR-confirmed clinical cases: 66 *P. falciparum*, 78 *P. vivax*, 126 *P. knowlesi*, 26 *P. malariae* and 10 *P. ovale* spp. (Table 1). There was a higher proportion of males in all malaria groups, most evident for knowlesi malaria cases (82.5%). Mean age was also highest for this species. Children with malaria (< 15 years) made up 6.3% to 34.6% across groups. A total of 25 patients were categorized as having severe malaria (3 *P. falciparum*, 1 *P. vivax*, 21 *P. knowlesi*). Median parasitemia was significantly higher in *P. falciparum* patients compared to other infecting species (Table 1; *P* < 0.0001). Plasma and a subset of corresponding whole blood samples (Table 1) were used to assess the diagnostic performance of the multiplex assay. There were no statistically significant differences in the distribution of parasitemias between the two sample types for each infecting species except for *P. knowlesi*, where parasitemia was significantly higher in the plasma than whole blood subset (median 1,590 vs 130 parasites/µL respectively; *P* = 0.005).

**Table 1:** Baseline characteristics.

	Healthy controls	PCR-confirmed species				
		*P. falciparum*	*P. vivax*	*P. knowlesi*	*P. malariae*	*P. ovale*
Sample size (n)	102	66	78	126	26	10
Gender (n of male [%])	45 (44)	49 (74.2)	56 (71.8)	104 (82.5)	19 (73.1)	5 (55.6)
Age (median [IQR])	31 (27–42)	20.5 (13–39)	18.5 (11.8–34)	37 (22.8–50)	17 (11.8–26.3)	30 (24–43.5)
Children, < 15 years (n [%])	0 (0)	15 (22.7)	20 (25.6)	8 (6.3)	9 (34.6)	1 (11.1)
Parasitemia, count/µL (median [IQR] [Range])	0 (0)	12,000 (1,860–31,000) [0, 0–694]	4,060 (2,090–8,170) [408–84,400]	1,590 (118–14,700) [0, 27–584]	1,380 (387–3,530) [71–12,600]	3,120 (1,280–4,150) [276–5,030]
Severe malaria (n [%])	0 (0)	3 (4.5)	1 (1.3)	21 (16.7)	0	0
Patients with plasma available (n [%])	102 (100)	66 (100)	77 (98.7)	126 (100)	26 (100)	10 (100)
Patients with whole blood available (n [%])	102 (100)	63 (95.5)	66 (84.6)	66 (52.4)	24 (92.3)	2 (20)

Demographic data missing for 1 *P. ovale* patient; parasitemia missing for 1 *P. knowlesi* patient.

*PCR* polymerase chain reaction, *IQR* interquartile-range.

### Malaria antigen and CRP distribution across *Plasmodium* species and controls

**Figure 1:**
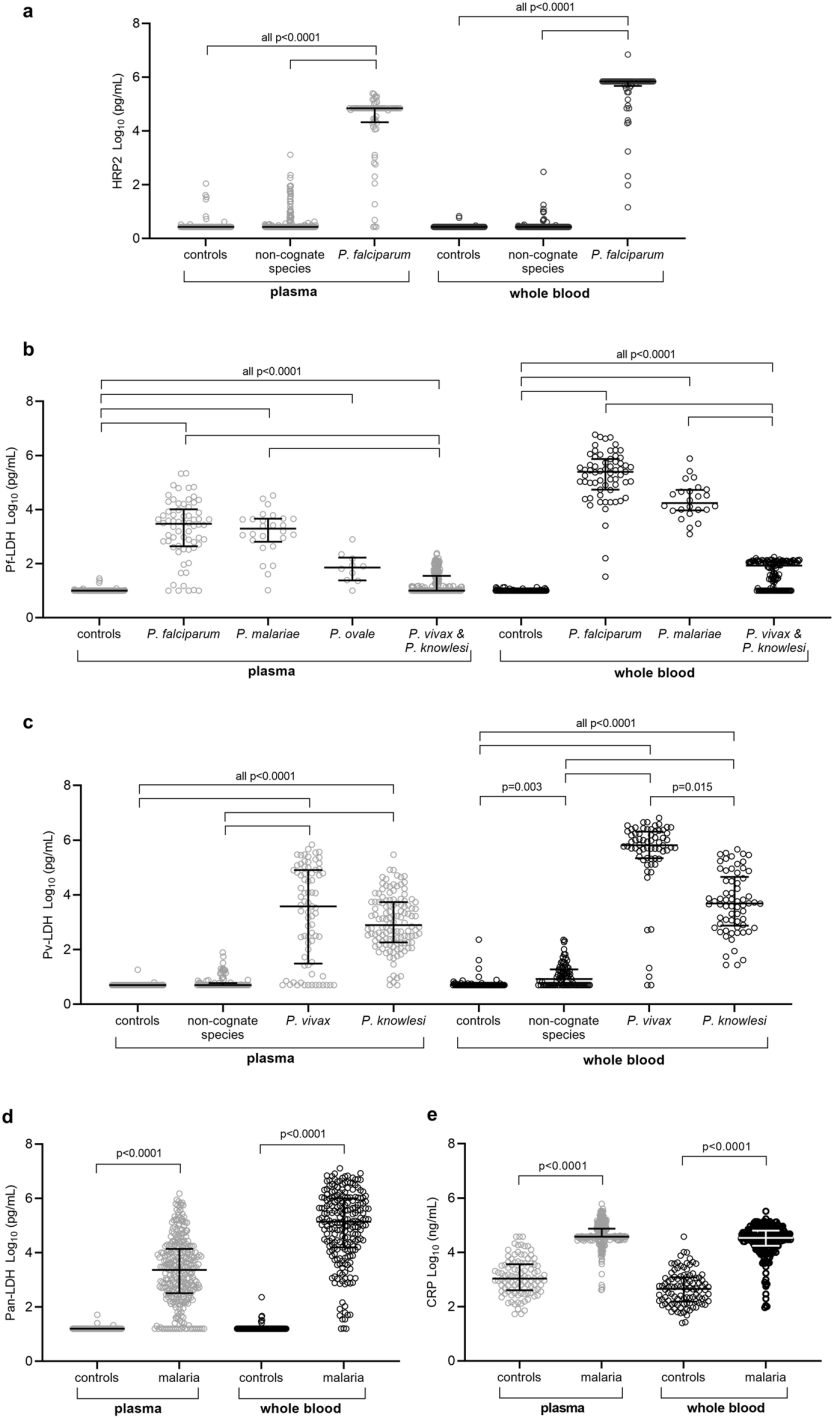
Distribution of malaria antigen concentrations in malaria patients and controls. Scatter plots of the concentration of histidine-rich protein-2 (**a**), *P. falciparum* lactate dehydrogenase (Pf-LDH) (**b**), *P. vivax* LDH (Pv-LDH) (**c**), *Pan* Plasmodium LDH (Pan-LDH) (**d**) and C-reactive protein (CRP) (**e**) were determined in plasma and whole blood samples. Refer to Table 1 for sample sizes. Non-cognate species in A correspond to Pv, *P. knowlesi* (Pk), *P. malariae* (Pm) and *P. ovale* (Po; plasma only); and in C correspond to Pf, Pm and Po. Antigen concentrations were log transformed. Individual datapoints with median and interquartile-range shown. The Kruskal–Wallis test with Dunn’s multiple comparison was used in (**a**)–(**c**), and the Man-Whitney test in (**d**) and (**e**). A *P*-value < 0.05 was considered statistically significant.

The Q-Plex™ Human Malaria assay measured whole blood and plasma concentrations of HRP2, pan-LDH, Pf-LDH, Pv-LDH and CRP. Overall, *Plasmodium* antigen concentrations were consistently higher in whole blood and generally showed less variability in concentration compared to plasma. For both sample types, HRP2 concentrations were significantly higher in falciparum malaria compared to controls or other malaria patients (Fig. 1a; *P* < 0.0001). The measured level of Pf-LDH was higher in samples from subjects infected with all *Plasmodium* species compared to controls, but was highest in patients with *P. falciparum* infection (Fig. 1b; *P* < 0.0001). For Pv-LDH, whole blood and plasma concentrations were markedly higher in vivax and knowlesi malaria (*P* < 0.0001), with whole blood concentrations also elevated in infections by other species compared to controls (Fig. 1c; *P* = 0.003). Concentrations of pan-LDH and CRP were significantly elevated in malaria patients compared to controls for both sample types (Fig. 1d,e; *P* < 0.0001).

### Sensitivity and specificity of HRP2 for *P. falciparum*

The assay’s diagnostic performance was evaluated against clinical samples with PCR-confirmed mono-infection of five different *Plasmodium* species (Table 2). Results for HRP2 showed excellent sensitivity and specificity (against controls) for *P. falciparum* infections in whole blood (100% and 98%, respectively). The diagnostic sensitivity of the HRP2 assay was less for plasma samples (92.4%), but specificity remained high (98%). The overall specificity of the HRP2 assay for *P. falciparum* compared against other *Plasmodium* species was 93% in whole blood and 94.6% in plasma.

**Table 2:** Q-Plex™ Human Malaria assay diagnostic performance characteristics.

Analyte	PCR-Confirmed species	n	Threshold (pg/mL)	Sensitivity (95%CI)	Specificity (95%CI)							
					vs controls	vs Pf	vs Pv	vs Pk	vs Pm	vs Po	vs all malaria	vs all malaria + controls
**Plasma**												
HRP2	Pf	66	40.84	92.4 (83.2–97.5)	98.0 (93.1–99.8)	-	97.4 (90.9–99.7)	91.3 (84.9–95.6)	100 (86.8–100)	100 (66.4–100)	94.6 (90.9–97.1)	95.6 (92.9–97.5)
Pf-LDH	Pf	66	17.81	89.4 (79.4–95.6)	97.1 (91.6–99.4)	-	61.0 (49.3–72.0)	69.8 (61.0–77.7)	3.9 (0.1–19.6)	10 (0.3–44.5)	57.3 (50.8–63.7)	69.2 (64.0–74.1)
Pf-LDH	Pm	26	17.81	96.2 (80.4–99.9)	As above	10.6 (4.4–20.6)	As above	As above	-	As above	51.3 (45.2–57.3)	63.5 (58.5–68.4)
Pf-LDH	Pk	126	17.81	30.2 (22.3–39.0)	As above	As above	As above	-	3.9 (0.1–19.6)	As above	31.3 (24.6–38.6)	55.2 (49.1–61.1)
Pv-LDH	Pv	77	9.14	80.5 (69.9–88.7)	99.0 (94.7–100)	83.3 (72.1–91.4)	-	3.2 (0.9–7.9)	88.5 (69.9–97.6)	80 (44.4–97.5)	39.5 (33.1–46.1)	57.9 (52.4–63.3)
Pv-LDH	Pk	126	9.14	96.8 (92.1–99.1)	As above	As above	19.5 (11.3–30.1)	-	As above	As above	56.4 (48.8–63.8	71.9 (66.2–77.1)
Pan-LDH	All malaria	305	27.56	90.2 (86.3–93.3)	99.0 (94.7–100)	-	-	-	-	-	-	-
CRP	All malaria	305	26,212^a^	86.6 (82.2–90.2)	97.1 (91.6–99.4)	-	-	-	-	-	-	-
**Whole blood**												
HRP2	Pf	63	4.31	100 (94.3–100)	98.0 (93.1–99.9)	-	92.4 (83.2–97.5)	93.9 (85.2–98.3)	95.8 (78.9–99.9)	-	93.0 (87.9–96.5)	95.0 (91.6–97.3)
Pf-LDH	Pf	63	12.64	100 (94.3–100)	96.1 (90.3–98.9)	-	10.6 (4.4–20.6)	47.0 (34.6–59.7)	0 (0–14.3)	-	24.1 (17.6–31.5)	52.3 (46.1–58.5)
Pf-LDH	Pm	24	12.64	100 (85.8–100)	As above	0 (0–5.7)	As above	As above	-	-	19.3 (14.0–25.5)	45.5 (39.7–51.3)
Pf-LDH	Pk	66	12.64	53.0 (40.3–65.4)	As above	As above	As above	-	0 (0–14.3)	-	4.5 (1.8–9.1)	40.9 (34.8–47.1)
Pv-LDH	Pv	66	75.66	93.9 (85.2–98.3)	99.0 (94.7–100)	93.7 (84.5–98.2)	-	6.1 (1.7–14.8)	100 (85.8–100)	-	57.4 (49.2–65.3)	73.9 (68.1–79.2)
Pv-LDH	Pk	66	75.66	93.9 (85.2–98.3)	As above	As above	6.1 (1.7–14.8)	-	as above	-	57.4 (49.2–65.3)	73.9 (68.1–79.2)
Pan-LDH	all malaria	221	84.42	95.5 (91.8–97.8)	99.0 (94.7–100)	-	-	-	-	-	-	-
CRP	all malaria	221	13,768^a^	80.1 (74.2–85.2)	99.0 (94.7–100)	-	-	-	-	-	-	-

*PCR* polymerase chain reaction, *n* number of samples, *CI* confidence interval, *Pf Plasmodium falciparum*, *Pv Plasmodium vivax*, *Pk Plasmodium knowlesi*, *Pm Plasmodium malariae*, *Po Plasmodium ovale*, *HRP2* Histidine-rich protein-2, *LDH* lactate dehydrogenase, *CRP* C-reactive protein.

^a^Units of ng/mL.

### Pf-LDH antibody cross reacts in *P. malariae* and *P. ovale* infection

Like HRP2, Pf-LDH showed excellent sensitivity for *P. falciparum* infection in whole blood (100%) but again was less sensitive in plasma (89.4%; Table 2). However, while its specificity against malaria negative controls was high (96–97%), there was significant cross reactivity of the Pf-LDH assay in infections with *P. malariae* and *P. ovale* reflected by the poor specificities against these species (3.9% and 10% in plasma, respectively, with an inability to differentiate between species in whole blood). In fact, the Pf-LDH assay was more sensitive for detection of *P. malariae* than *P. falciparum* in plasma samples (96.2%) and detected 100% of *P. malariae* cases in whole blood. The specificity of Pf-LDH for detection of *P. falciparum* was similarly low compared to *P. vivax* (10.6%) and *P. knowlesi* (47.0%) infections in whole blood, improving to 61.0% and 69.8% in plasma, respectively. The sensitivity of Pf-LDH in detecting *P. vivax* and *P. knowlesi* infections was 89.4% and 53.0% in whole blood, and 39.0% and 30.2% in plasma, respectively. In the two cases of *P. ovale* where whole blood was available, there were strong positive signals for Pf-LDH (5,358 and 197 pg/mL) and pan-LDH (10,723 and 1,697 pg/mL), while measures of other *Plasmodium* antigens were below threshold. Therefore, while Pf-LDH is highly sensitive in detecting *P. falciparum* infections in whole blood, our results indicate that Pf-LDH alone displays limited specificity and requires an alternate analyte such as HRP2 for specific diagnosis of *P. falciparum* infection in co-endemic regions. Given that this may be problematic in cases where *hrp2/3* gene deletions are prevalent, we calculated the pan-LDH-to-Pf-LDH ratio in whole blood and found that 97% of *P. falciparum* cases had ratios < 1.5 (median 0.75 [IQR:0.69–0.91]) while 92% of *P. malariae* and *P. ovale* cases had ratios > 1.5 (median 2.74 [IQR:2.53–3.41], *P* < 0.0001; Supplementary Fig S1a). Thus, the pan-LDH-to-Pf-LDH ratio may offer a strategy to diagnose *P. falciparum* infections in the absence of a positive HRP2 antigen result.

### Pv-LDH antibody cross reacts in *P. knowlesi* infection

The sensitivity of the Pv-LDH test in whole blood and plasma was suboptimal for *P. vivax* infections (93.9% and 80.5%, respectively; Table 2), and there was high level cross-reactivity of Pv-LDH in PCR-confirmed *P. knowlesi* infections, with the Pv-LDH assay displaying better performance at detecting *P. knowlesi* (93.9% and 96.8% sensitivity in whole blood and plasma, respectively). The specificity of the Pv-LDH assay for *P. vivax* and *P. knowlesi* infection in whole blood was high (99% versus controls, 93.7% versus *P. falciparum* and 100% versus *P. malariae* and *P. ovale*), but specificity was lower in infection with other species in plasma samples (80–88.5%). The pan-LDH-to-Pv-LDH ratio did not distinguish *P. vivax* from *P. knowlesi* infection. However, the pan-LDH-to-Pf-LDH ratio in whole blood was above a threshold of 3,000 in 88% of *P. vivax* cases (median 14,300 [IQR:7,420–32,600), while 83% of *P. knowlesi* cases had ratios < 3,000 (median 323 [IQR:102–2,050], *P* < 0.0001; Supplementary Fig. S1b). Taken together, the assay’s measurement of whole blood Pv-LDH allows reasonably accurate identification of *P. vivax* or *P. knowlesi* infections but alone cannot distinguish between the two parasites where both *Plasmodium* species co-exist.

### Performance of pan-LDH

The sensitivity and specificity of pan-LDH was assessed using all malaria patients as a single group (Table 2). Pan-LDH showed high sensitivity as a marker to diagnose any malaria in whole blood (211/221; 95.5%) and showed high specificity in both sample types (99%).

### Paired comparison of whole blood versus plasma

The performance of the assay in plasma samples was compared to matched whole blood samples (Table 3). In whole blood, 100% of *P. falciparum* cases were detected with targets HRP2 and Pf-LDH, significantly higher than the proportion of cases detected in matching plasma (92.1% and 88.9%, respectively; *P* < 0.05). Likewise, the diagnostic sensitivity of Pv-LDH at detecting *P. vivax* cases was significantly higher in whole blood (93.9%) than in plasma (76.9%, *P* = 0.002), while its sensitivity for *P. knowlesi* infections was similar in the two sample matrices (93.9% versus 97%, *P* = 0.54). There was no significant difference in Pf-LDH sensitivity for *P. malariae* infections between whole blood (100%) and plasma (95.8%, *P* = 0.62). Collectively, the assay showed superior diagnostic performance using the recommended sample type of whole blood, and adequate performance in plasma. In contrast, the host-response protein, CRP, was elevated in significantly more malaria cases when using plasma (89.6%) compared to whole blood (80%).

**Table 3:** Comparison of assay sensitivity in paired plasma and whole blood.

Analyte	PCR-confirmed species	n of paired samples	Plasma		Whole blood		P-value from McNemar’s paired test comparing plasma vs whole blood sensitivity	
			Threshold (pg/mL)	Sensitivity (95%CI)	Threshold (pg/mL)	Sensitivity (95%CI)	Uncorrected	Yates correction
HRP2	Pf	63	40.84	92.1 (82.4–97.4)	4.31	100 (94.3–100)	0.025	0.044
Pf-LDH	Pf	63	17.81	88.9 (78.4–95.4)	12.64	100 (94.3–100)	0.008	0.014
Pf-LDH	Pm	24	17.81	95.8 (78.9–99.9)	12.64	100 (85.8–100)	0.32	0.62
Pv-LDH	Pv	65	9.14	76.9 (64.8–86.5)	75.66	93.9 (85.0–98.3)	0.0009	0.002
Pv-LDH	Pk	66	9.14	97.0 (89.5–99.6)	75.66	93.9 (85.2–98.3)	0.41	0.54
Pan-LDH	All malaria	220	27.56	87.3 (82.1–91.4)	84.42	95.5 (91.8–97.8)	0.0001	0.0002
CRP	All malaria	220	26,212^a^	89.6 (84.7–93.3)	13,768.^a^	80.0 (74.1–85.1)	0.0006	0.0008

*PCR* polymerase chain reaction, *CI* confidence interval, *Pf* *Plasmodium falciparum*, *Pv* *Plasmodium vivax*, *Pk* *Plasmodium knowlesi*, *Pm* *Plasmodium malariae*, *Po* *Plasmodium ovale*, *HRP2* Histidine-rich protein-2, *LDH* lactate dehydrogenase, *CRP* C-reactive protein.

^a^Units of ng/mL.

### Choice of threshold cut-offs for positivity

For all 5 antigens, we reanalysed the data using two separate cut-off thresholds for positivity derived from ROC curves (Supplementary Table S1). A summary of the area under ROC curves is shown in Supplementary Table S2. Thresholds giving > 99% specificity on ROC curves versus controls resulted in similar diagnostic performance to those determined using our default method. In contrast, improving the specificity by applying higher thresholds that gave > 99% specificity on ROC curves versus non-cognate *Plasmodium* species, resulted in lower sensitivities for all species except for *P. vivax* and *P. malariae* detection using whole blood Pv-LDH and Pf-LDH, respectively (Supplementary Table S1). Using these alternative thresholds in the paired comparison of sample types, the diagnostic performance using whole blood remained superior compared to plasma (Supplementary Table S3). Taken together, our data indicate that slight variability in control-based threshold cut-offs do not significantly alter the assay’s performance, which can be tailored to the setting or sample-set being evaluated.

### Parasite counts in malaria samples with undetectable parasite antigens

We evaluated the range of peripheral blood parasite concentrations in samples where parasite antigen levels were below the detection threshold in whole blood and plasma (Supplementary Tables S4). HRP2 and Pf-LDH were detectable in whole blood in all *P. falciparum* infections, including at the lowest reported parasitemia of 34 parasites/µL. In *P. vivax* infections, parasite counts of up to 16,100 parasites/µL were not detected with the Pv-LDH antibody in whole blood. Thus, applying a high parasitemia cut-off of 2,000 parasites/µL, as recommended by World Health Organization for assessment of pLDH-based RDTs^29^, did not improve the sensitivity of Pv-LDH for *P. vivax* infections. In contrast, only at very low parasitemias (≤ 105 parasites/µL) did knowlesi malaria samples test negative using the Pv-LDH antibody in whole blood, which were irrelevant when applying the lower recommended WHO cut-off of 200 parasites/µL^29^. Pf-LDH antibodies detected all *P. malariae* infections, including parasitemias as low as 71 parasites/µL.

## Discussion

Our study evaluated the diagnostic performance of the Q-Plex™ Human Malaria assay against a set of 306 PCR-confirmed cases of malaria mono-infection with five different *Plasmodium* species, including clinical *P. knowlesi* samples, and provide new data comparing its diagnostic performance in whole blood versus plasma samples for all species. Our results indicate that this platform has excellent performance when *P. falciparum* HRP2 is the target, but when Pf-LDH antigen is the target there is significant cross-reactivity with *P. malariae* and *P. ovale* (and weaker cross-reactivity with *P. vivax* and *P. knowlesi*). The inclusion of Pv-LDH in the assay panel allowed the detection of *P. vivax* cases with 94% sensitivity. However, the Pv-LDH target could result in misdiagnosis of *P. knowlesi* infections in co-endemic areas due to very high cross-reactivity with this species, as demonstrated by the excellent reported sensitivity for *P. knowlesi*. This is not surprising as there are only five amino acid polymorphisms between *P. vivax* and *P. knowlesi* in the 316 residues of the LDH protein. Examination of whole blood demonstrated superior diagnostic performance compared to plasma for the majority of antigens. Overall, our study further supports the Quansys 5-plex assay as a promising platform for malaria diagnostic research and development but, in its current form and price per specimen (~ 20 Australian dollars), may be limited to high-throughput usage in well-funded reference laboratories in selected malaria-endemic settings.

A different approach to the determination of threshold cut-offs for positivity (values giving 98.5% and 99.5% specificity against controls) was used in previous evaluations^7,8^. Here, we opted to set a different threshold (mean plus 3 × SD of controls) and found this did not significantly alter the diagnostic performance of the assay. In a clinical or surveillance setting, the expected profile for malaria diagnostics includes a minimum sensitivity and specificity of 95%, and for optimum performance a sensitivity of 98% and specificity of 99%^30^. The Quansys assay met these criteria for the detection of *P. falciparum* infections due to the excellent performance of the HRP2-detecting component. HRP2 positivity in a small proportion of non-falciparum species was possibly a reflection of recent *P. falciparum* infection and the longer persistence of HRP2 after treatment and parasite clearance^31^. Importantly, we found that the assay requires further development for precise identification of non-falciparum species. The high level conservation of the LDH protein among the different *Plasmodium* species, with resultant cross-reacting antibody specificity, means that the Pf-LDH cannot distinguish *P. falciparum* from *P. malariae*^7^ and *P. ovale*, and is consistent with previous findings^10^. Despite this, it was possible to differentiate *P. malariae* and *P. ovale* from *P. falciparum* by using the lack of HRP2 positivity. In regions with high rates of *hrp2/3* gene deletions where *P. falciparum* and *P. malariae/ovale* may co-exist^32,33^, using the whole blood pan-LDH-to-Pf-LDH ratio would allow distinction of *P. falciparum* infections (ratio < 1.5) from the two cross-reacting species (ratio > 1.5).

In addition to demonstrating known *P. knowlesi* cross-reactivity with Pf-LDH^17,19^, the platform’s diagnostic performance for *P. knowlesi* revealed strong cross reactivity with the *P. vivax* marker (Pv-LDH) as previously reported with both cultured A1H1 strain samples^10^ and using other diagnostic methods^18,34,35^. The superior sensitivity of RDTs targeting Pv-LDH in detecting *P. knowlesi* over *P. vivax* has previously been reported^35^ and was seen in our plasma samples. We were unable to explain the 4 false negative *P. vivax* cases with relatively high parasitemia. Genetic diversity of the protein ^36^ or sample damage in these cases are unlikely, and testing of these cases at higher whole blood dilutions did not improve detection, excluding any inhibitory effects as a cause. The pan-LDH-to-Pf-LDH ratio may be a strategy to differentiate *P. vivax* from *P. knowlesi* infection but with some uncertainty. While the sensitivity and specificity of Pv-LDH to detect these two species in whole blood (~ 94%) fell short of the expected diagnostic profiles (≥ 95%), it may be useful in areas endemic for *P. knowlesi* without or with very low *P. vivax* prevalence such as Malaysia, or in areas endemic for *P. vivax* with very low *P. knowlesi* prevalence. The development of diagnostic tools to robustly distinguish *P. vivax* from *P. knowlesi* infection is urgently needed to support clinical management and public health responses. In co-endemic areas, misdiagnosing knowlesi malaria as vivax infection may result in suboptimal oral treatment of potentially life-threatening knowlesi infection which may require immediate parenteral artesunate therapy^20,37,38^. Conversely, it may result in unnecessary primaquine therapy in knowlesi malaria and complicate public health responses for elimination of human-only *Plasmodium* infections, while misdiagnosis of vivax malaria as *P. knowlesi* infection would result in failure to administer primaquine to prevent relapse.

Pan-LDH was a useful additional marker in the assay. It has utility for calculation of the pan-LDH-to-Pf-LDH ratio to discriminate *P. falciparum* from *P. malariae/ovale* in the absence of HRP2. Inclusion of CRP has been argued to be of prognostic value^39,40^. We were unable to assess the utility of CRP in predicting severe malaria in our study due to low numbers of severe malaria cases with whole blood samples available (n = 5). Further, without additional testing to exclude potential concomitant bacterial infections that may contribute to raised CRP^41^, it was not possible to the infer the utility of this biomarker for assessment of malaria-only severity. Moreover, as assay dilutions beyond 100-fold did not allow identification of exact concentrations in samples with very high CRP levels, we were unable to quantitatively assess for this relationship. A limitation of this assay platform, where a multiplex approach is used, is that the optimal dilution for each analyte may not be achieved, and rerunning samples at multiple dilutions may not be possible, for example where sample volumes are limited. Other diagnostic limitations of the assay include duration of the assay (~ 4 h – similar length as PCR), high cost, and the need for an imager and power, overall restricting the current Quansys platform for use in larger facilities.

Greater diagnostic sensitivity of *Plasmodium* antigens in whole blood compared to plasma samples (though not statistically significant in *P. knowlesi* and *P. malariae*) was expected given greater antigen quantity released from lysed red cells than that present in plasma. The greater variation in *Plasmodium* antigen concentrations seen in plasma versus whole blood, together with recent observations comparing native versus recombinant species-specific pLDH using this platform^10^, also point towards differences in antibody-binding to target pLDH epitopes. Conformational changes of the target protein in plasma and/or a higher proportion of immune-complexed protein in plasma from humoral immunity^42^, may also contribute to greater sensitivity in whole blood. As a diagnostic tool, whole blood is a preferred analyte, given the extra work and instrumentation needed to separate plasma. Scenarios where the latter may be necessary include serological surveillance surveys, retrospective studies where whole blood is not available, or analytical studies evaluating protein secretion and biomarkers of parasite biomass^43,44^.

In summary, our findings support the Q-Plex™ Human Malaria assay as a sensitive tool for the diagnosis of symptomatic malaria from all five major *Plasmodium* species^7,8^. In its current configuration as an ELISA assay with imager, suitable applications include a reference assay for RDT quality assurance, research and development and potentially at reference laboratories where simultaneous testing of large numbers of samples is required. Further optimisation of the assay platform will be required to improve specificity for diagnosis of non-falciparum species if it is to be considered a platform for evaluating biomarkers for expansion into next-generation RDTs. Moreover, the study reiterates the limitations of current reagents developed to detect pLDH and perhaps the suitability of LDH as a target analyte to selectively discriminate non-falciparum malaria species.

## Supplementary Information

Supplementary Information.

## Data Availability

The data in this manuscript is available from the authors upon reasonable request.
