# Supplementary material for: Diagnostic performance of a 5-plex malaria immunoassay in regions co-endemic for Plasmodium falciparum, P. vivax, P. knowlesi, P. malariae and P. ovale
Source: Sci Rep. 2022 May 4;12:7286. doi: 10.1038/s41598-022-11042-w (PMC9068623; doi:10.1038/s41598-022-11042-w)
Supplement: Supplementary file 1 — Supplementary Information. [file 41598_2022_11042_MOESM1_ESM.pdf]

# Supplementary Materials

**Title:** Diagnostic performance of a 5-plex malaria immunoassay in regions co-endemic for *Plasmodium falciparum*, *P. vivax*, *P. knowlesi*, *P. malariae* and *P. ovale*


## Authors

Steven Kho[a*], Nicholas M. Anstey[a], Bridget E. Barber[a,b], Kim Piera[a], Timothy William[c], Enny Kenangalem[d], James S McCarthy[e], Ihn Kyung Jang[f], Gonzalo J. Domingo[f], Sumudu Britton[b#], Matthew J. Grigg[a#].

#equal contribution

## Affiliation

[a]Menzies School of Health Research, Darwin, NT, Australia

[b]QIMR-Berghofer Medical Research Institute, Brisbane, QLD, Australia

[c]Clinical Research Centre, Queen Elizabeth Hospital, Kota Kinabalu, Malaysia

[d]Papuan Health and Community Development Foundation, Timika, Indonesia

[e]The Peter Doherty Institute for Infection and Immunity, University of Melbourne and Royal Melbourne Hospital, Melbourne, VIC, Australia

[f]Diagnostic Program, PATH, Seattle, USA.

*Corresponding author name: Steven Kho

Corresponding author e-mail: steven.kho@menzies.edu.au

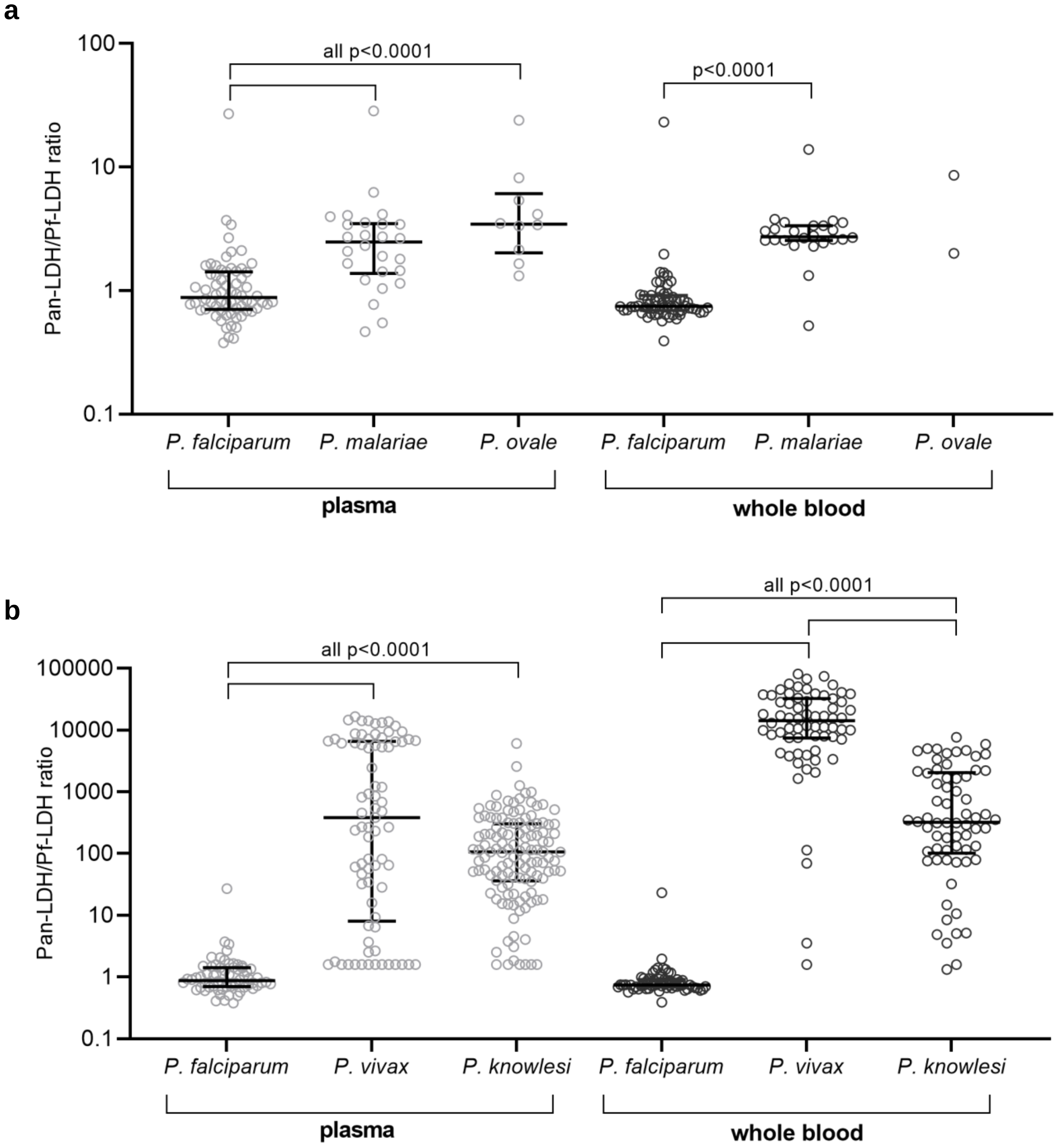


**Supplementary Figure S1. Pan-LDH/Pf-LDH ratio to differentiate *P. falciparum* from *P. malariae* and *P. ovale,* and *P. vivax* from *P. knowlesi*.** Scatter plots of the pan *Plasmodium* lactate dehydrogenase (Pan-LDH)-*to-P. falciparum* LDH (Pf-LDH) ratio in falciparum, malariae and ovale malaria patients (A), and for falciparum, vivax and knowlesi malaria patients (B). Refer to Table 1 for sample sizes. Individual datapoints with median and interquartile-range shown. The Kruskal-Wallis test with Dunn’ s multiple comparison was used with a p-value <0.05 considered statistically significant.

**Supplementary Table S1. Assay performance characteristics using threshold cut-off values giving >99% specificity on ROC curve vs controls or non-cognate species**

| Analyte | PCR-confirmed species | Positivity threshold derived from | Threshold (pg/mL) | Sensitivity (95%CI) | Specificity (95%CI) | | | | | | | |
|---|---|---|---|---|---|---|---|---|---|---|---|---|
| | | | | | vs controls | vs Pf | vs Pv | vs Pk | vs Pm | vs Po | vs all malaria | vs all malaria + controls |
| **Plasma** | | | | | | | | | | | | |
| HRP2 | Pf | controls | 76.24 | 92.4 (83.2-97.5) | 99.0 (94.7-100) | - | 97.4 (90.9-99.7) | 96.0 (91.0-98.7) | 100 (86.8-100) | 100 (69.2-100) | 97.1 (94.1-98.8) | 97.7 (95.4-100) |
| HRP2 | Pf | non-cognate: Pv, Pk, Pm, Po | 403.70 | 89.4 (79.4-95.6) | 100 (96.5-100) | - | 100 (95.3-100) | 99.2 (95.7-100) | 100 (86.8-100) | 100 (69.2-100) | 99.6^ (97.7-100) | 99.7 (98.4-100) |
| Pf-LDH | Pf | controls | 25.98 | 89.4 (79.4-95.6) | 99.02 (94.7-100) | - | 68.8 (57.3-78.9) | 71.4 (62.7-79.1) | 3.9 (0.1-19.6) | 30.0 (6.7-65.3) | 61.5 (55.0-67.7) | 72.7 (67.7-77.4) |
| Pf-LDH | Pf | non-cognate: Pv, Pk | 231.9 | 83.3 (72.1-91.4) | 100 (96.5-100) | - | 98.7 (83.0-100) | 100 (97.1-100) | 15.4 (4.4-34.9) | 90.0 (55.5-99.8) | 99.5^ (97.3-100) | 93.0 (89.7-95.4) |
| Pf-LDH | Pm | controls | 25.98 | 96.2 (80.4-99.9) | 99.02 (94.7-100) | 10.6 (4.4-20.6) | 68.8 (57.3-78.9) | 71.4 (62.7-79.1) | - | 30.0 (6.7-65.3) | 54.8 (48.8-60.8) | 66.7 (61.7-71.4) |
| Pf-LDH | Pm | non-cognate: Pv, Pk | 231.9 | 84.6 (65.1-95.6) | 100 (96.5-100) | 16.7 (8.6-27.9 | 98.7 (83.0-100) | 100 (97.1-100) | - | 90.0 (55.5-99.8) | 99.5^ (97.3-100) | 85.0 (81.1-88.5) |
| Pv-LDH | Pv | controls | 6.24 | 83.1 (72.9-90.7) | 99.02 (94.7-100) | 74.2 (62.0-84.2) | - | 1.6 (0.2-5.6) | 88.5 (69.9-97.6) | 80 (44.4-97.5) | 36.0 (29.7-42.6) | 55.5 (49.9-60.9) |
| Pv-LDH | Pv | non-cognate: Pf, Pm, Po | 90.0 | 72.7 (61.4-82.3) | 100 (96.5-100) | 100 (94.6-100) | - | 13.5 (8.1-20.7) | 100 (86.8-100) | 100 (69.2-100) | 100^ (96.5-100) | 67.0 (61.6-72.0) |
| Pv-LDH | Pk | controls | 6.27 | 98.4 (94.4-99.8) | 99.0 (94.7-100) | 74.2 (62.0-84.2) | 18.2 (10.3-28.6) | - | 88.5 (69.9-97.6) | 80.0 (44.4-97.5) | 52.5 (44.9-60.0) | 69.4 (63.6-74.7) |
| Pv-LDH | Pk | non-cognate: Pf, Pm, Po | 56.42 | 92.1 (85.89-96.13) | 100 (96.5-100) | 100 (94.6-100) | 27.3 (17.7-38.6) | - | 100 (86.8-100) | 90.0 (55.5-99.8) | 99.0^ (94.7-100) | 79.7 (74.5-84.3) |
| Pan-LDH | all malaria | controls | 26.16 | 90.5 (86.6-93.5) | 99.0 (94.7-100) | - | - | - | - | - | - | - |
| CRP | all malaria | controls | 21,163* | 89.5 (85.5-92.7) | 97.1 (91.6-99.4) | - | - | - | - | - | - | - |
| **Whole blood** | | | | | | | | | | | | |
| HRP2 | Pf | controls | 6.35 | 100 (94.3-100) | 99.0 (94.7-100) | - | 97.0 (89.5-99.6) | 93.9 (85.2-98.3) | 100 (85.8-100) | - | 96.2 (91.9-98.6) | 97.3 (94.5-98.9) |
| HRP2 | Pf | non-cognate: Pv, Pk, Pm, Po | 57.15 | 98.4 (91.5-100) | 100 (96.5-100) | - | 100 (94.6-100) | 98.5 (91.8-100) | 100 (85.8-100) | - | 99.4^ (96.5-100]) | 99.6 (97.9-100) |
| Pf-LDH | Pf | controls | 13.54 | 100 (94.3-100) | 99.0 (94.7-100) | - | 10.6 (4.4-20.6) | 47.0 (34.6-59.7) | 0 (0-14.3) | - | 24.1 (17.6-31.5) | 53.5 (47.2-59.7) |
| Pf-LDH | Pf | non-cognate: Pv, Pk | 158.7 | 98.4 (91.5-100) | 100 (96.5-100) | - | 100 (94.6-100) | 98.5 (91.8-100) | 0 (0-14.3) | - | 99.2^ (95.9-100) | 89.6 (85.3-93.0) |
| Pf-LDH | Pm | controls | 13.54 | 100 (85.8-100) | 99.0 (94.7-100) | 0 (0-5.7) | 10.6 (4.4-20.6) | 47.0 (34.6-59.7) | - | - | 19.3 (14.0-25.5) | 46.5 (40.7-52.3) |
| Pf-LDH | Pm | non-cognate: Pv, Pk | 166.9 | 100 (85.8-100) | 100 (96.5-100) | 3.2 (0.4-11.0) | 100 (94.6-100) | 98.5 (91.8-100) | - | - | 99.2^ (95.9-100) | 78.6 (73.5-83.1) |
| Pv-LDH | Pv | controls | 135.80 | 93.9 (85.2-98.3) | 99.0 (94.7-100) | 95.3 (86.7-99.0) | - | 7.6 (2.5-16.8) | 100 (85.8-100) | - | 58.7 (50.5-66.6) | 74.7 (68.9-79.9) |

| | | | | | | | | | | | | |
|---|---|---|---|---|---|---|---|---|---|---|---|---|
| Pv-LDH | Pv | non-cognate: Pf, Pm, Po | 219.9 | 93.9 (85.2-98.3) | 99.0 (94.7-100) | 98.4 (91.5-100) | - | 7.6 (2.5-16.8) | 100 (85.8-100) | - | 98.9^ (93.9-100) | 72.8 (66.9-78.1) |
| Pv-LDH | Pk | controls | 48.24 | 95.5 (87.3-99.1) | 99.0 (94.7-99.100) | 87.3 (76.5-94.4) | 6.1 (1.7-14.8) | - | 100 (85.8-100) | - | 54.8 (46.7-62.8) | 72.4 (66.5-77.8) |
| Pv-LDH | Pk | non-cognate: Pf, Pm, Po | 219.9 | 92.4 (83.2-97.5) | 99.0 (94.7-99.100) | 98.4 (91.5-100) | 6.1 (1.7-14.8) | - | 100 (85.8-100) | - | 98.9^ (93.9-100) | 77.0 (71.4-82.0) |
| Pan-LDH | all malaria | controls | 47.88 | 97.7 (94.8-99.3) | 99.0 (94.7-99.100) | - | - | - | - | - | - | - |
| CRP | all malaria | controls | 10,167* | 85.5 (80.2-89.9) | 99.0 (94.7-99.100) | - | - | - | - | - | - | - |

Footnotes:

*units of ng/mL.

^against non-cognate species only

Refer to Table 1 for sample sizes.

Abbreviations: PCR, Polymerase chain reaction; CI, confidence interval; Pf, *Plasmodium falciparum*; Pv, *Plasmodium vivax*; Pk, *Plasmodium knowlesi*; Pm, *Plasmodium malariae*; Po, *Plasmodium ovale*; HRP2, Histidine-rich protein-2; LDH, lactate dehydrogenase; CRP, C-reactive protein.

## Supplementary Table S2. Area under ROC curves

| Analyte | PCR-confirmed species | Area under ROC curve (95% confidence interval) | | | |
|---|---|---|---|---|---|
| | | Plasma | | Whole blood | |
| | | vs controls | vs non-cognate species | vs controls | vs non-cognate species |
| HRP2 | Pf | 0.98 (0.96-1) | 0.97 (0.95-1) | 1 | 0.99 (0.99-1) |
| Pf-LDH | Pf | 0.97 (0.93-1) | 0.93 (0.88-0.98) | 1 | 0.99 (0.97-1) |
| Pf-LDH | Pm | 0.99 (0.99-1) | 0.97 (0.93-1) | 1 | 1 |
| Pv-LDH | Pv | 0.93 (0.88-0.87) | 0.89 (0.83-0.94) | 0.98 (0.96-1) | 0.97 (0.93-1) |
| Pv-LDH | Pk | 0.99 (0.98-1) | 0.98 (0.97-1) | 0.99 (0.99-1) | 0.99 (0.98-1) |
| Pan-LDH | all malaria | 0.96 (0.94-0.98) | - | 0.99 (0.99-1) | - |
| CRP | all malaria | 0.97 (0.95-0.99) | - | 0.97 (0.95-0.99) | - |

Footnotes:

Abbreviations: ROC, receiver operating characteristic; Pf, *Plasmodium falciparum*; Pv, *Plasmodium vivax*; Pk, *Plasmodium knowlesi*; Pm, *Plasmodium malariae*; HRP2, Histidine-rich protein-2; LDH, lactate dehydrogenase; CRP, c-reactive protein.

**Supplementary Table S3. Comparison of assay sensitivity in paired plasma and whole blood using threshold cut-off values giving >99% specificity on ROC curve vs controls or non-cognate species**

| Analyte | PCR-confirmed species | n of paired samples | Positivity threshold derived from | Plasma | | Whole blood | | P-value from McNemar's paired test comparing plasma vs whole blood sensitivity | |
|---|---|---|---|---|---|---|---|---|---|
| | | | | Threshold (pg/mL) | Sensitivity (95%CI) | Threshold (pg/mL) | Sensitivity (95%CI) | Uncorrected | Yates correction |
| HRP2 | Pf | 63 | controls | 76.24 | 92.1 (82.4-97.4) | 6.35 | 100 (94.3-100) | 0.025 | 0.044 |
| HRP2 | Pf | 63 | non-cognate: Pv, Pk, Pm, Po | 403.70 | 88.9 (78.4-95.4) | 57.15 | 98.4 (91.5-100) | 0.014 | 0.025 |
| Pf-LDH | Pf | 63 | controls | 25.98 | 88.9 (78.4-95.4) | 13.54 | 100 (94.3-100) | 0.008 | 0.014 |
| Pf-LDH | Pf | 63 | non-cognate: Pv, Pk | 231.9 | 82.5 (70.9-91.0) | 158.7 | 98.4 (91.5-100) | 0.002 | 0.003 |
| Pf-LDH | Pm | 24 | controls | 25.98 | 95.8 (78.9-99.9) | 13.54 | 100 (85.8-100) | 0.32 | 0.62 |
| Pf-LDH | Pm | 24 | non-cognate: Pv, Pk | 231.9 | 83.3 (62.6-95.3) | 166.9 | 100 (85.8-100) | 0.046 | 0.080 |
| Pv-LDH | Pv | 65 | controls | 6.24 | 80.0 (68.2-88.9) | 135.80 | 93.9 (85.0-98.3) | 0.007 | 0.010 |
| Pv-LDH | Pv | 65 | non-cognate: Pf, Pm, Po | 66.64 | 67.7 (54.9-78.8) | 219.9 | 93.9 (85.0-98.3) | <0.0001 | <0.0001 |
| Pv-LDH | Pk | 66 | controls | 6.27 | 100 (94.6-100) | 48.24 | 95.5 (87.3-99.1) | 0.083 | 0.15 |
| Pv-LDH | Pk | 66 | non-cognate: Pf, Pm, Po | 56.42 | 89.4 (79.4-95.6) | 219.9 | 92.4 (83.2-97.5) | 0.53 | 0.64 |
| Pan-LDH | all malaria | 220 | controls | 26.16 | 87.7 (82.7-91.8) | 47.88 | 97.7 (94.8-99.3) | <0.0001 | <0.0001 |
| CRP | all malaria | 220 | controls | 21,948* | 91.4 (86.8-94.7) | 10,167 | 85.5 (80.1-89.8) | 0.016 | 0.020 |

Footnotes:

*units of ng/mL.

Abbreviations: PCR, Polymerase chain reaction; CI, confidence interval; Pf, *Plasmodium falciparum*; Pv, *Plasmodium vivax*; Pk, *Plasmodium knowlesi*; Pm, *Plasmodium malariae*; Po, *Plasmodium ovale*; HRP2, Histidine-rich protein-2; LDH, lactate dehydrogenase; CRP, C-reactive protein.

**Supplementary Table S4. Summary of parasitemias below Quansys thresholds in *P. falciparum*, *P. vivax*, *P. knowlesi* and *P. malariae* infections**

| Parasitemia characteristics | PCR-confirmed Species | Analyte and sample type | | | | | | | |
|---|---|---|---|---|---|---|---|---|---|
| | | HRP2 | | Pf-LDH | | Pv-LDH | | Pan-LDH | |
| | | Plasma | WB | Plasma | WB | Plasma | WB | Plasma | WB |
| n of patients with parasitemia below threshold (n/N [%]) | Pf | 5/66 (7.6) | 0/63 (0) | 7/66 (10.6) | 0/63 (0) | - | - | 5/66 (7.6) | 1/63 (1.6) |
| | Pv | - | - | - | - | 15/77 (19.5) | 4/66 (6.1) | 16/77 (20.8) | 2/66 (3.0) |
| | Pk | - | - | - | - | 4/125 (3.2) | 4/66 (6.1) | 8/125 (6.4) | 7/66 (10.6) |
| | Pm | - | - | 1/26 (3.8) | 0/24 (0) | - | - | 1/26 (3.8) | 0/24 (0) |
| Median parasitemia below threshold, count/µL (range) | Pf | 1,480 (0-33,200) | 0 | 1,460 (34-15,200) | 0 | - | - | 1,460 (34-15,200) | 34 |
| | Pv | - | - | - | - | 3,740 (408-16,100) | 7,360 (1,230-16,100) | 3,630 (408-16,100) | 1,230; 16,100 |
| | Pk | - | - | - | - | 1,080 (66-8,540) | 88 (51-105) | 69 (51-8,540) | 66 (51-105) |
| | Pm | - | - | 3,056 | 0 | - | - | 3,056 | 0 |

Footnotes:

Refer to Table 1 for the parasitemia characteristics of total patients in each species group.

Abbreviations: Pf, *Plasmodium falciparum*; Pv, *Plasmodium vivax*; Pk, *Plasmodium knowlesi*; Pm, *Plasmodium malariae*; HRP2, Histidine-rich protein-2; LDH, lactate dehydrogenase; WB, whole blood.
